# Native *T*_1_ Mapping and Magnetization Transfer Imaging in Grading Bowel Fibrosis in Crohn’s Disease: A Comparative Animal Study

**DOI:** 10.3390/bios11090302

**Published:** 2021-08-28

**Authors:** Baolan Lu, Jinjiang Lin, Jinfang Du, Shaofu He, Qinghua Cao, Li Huang, Ren Mao, Canhui Sun, Ziping Li, Shiting Feng, Xuehua Li

**Affiliations:** 1 Department of Radiology, The First Affiliated Hospital, Sun Yat-sen University, Guangzhou 510080, China; lublan@mail.sysu.edu.cn (B.L.); linjinj@mail.sysu.edu.cn (J.L.); dujf3@mail2.sysu.edu.cn (J.D.); heshf6@mail.sysu.edu.cn (S.H.); huangli23@mail.sysu.edu.cn (L.H.); sunch@mail.sysu.edu.cn (C.S.); liziping@mail.sysu.edu.cn (Z.L.); 2 Department of Pathology, The First Affiliated Hospital, Sun Yat-sen University, Guangzhou 510080, China; caoqhua@mail.sysu.edu.cn; 3 Department of Gastroenterology, The First Affiliated Hospital, Sun Yat-sen University, Guangzhou 510080, China; maor5@mail.sysu.edu.cn

**Keywords:** Crohn’s disease, fibrosis, *T*_1_ mapping, magnetization transfer imaging

## Abstract

In this study, we investigated the utility of native *T*_1_ mapping in differentiating between various grades of fibrosis and compared its diagnostic accuracy to magnetization transfer imaging (MTI) in a rat model of CD. Bowel specimens (64) from 46 CD model rats undergoing native *T*_1_ mapping and MTI were enrolled. The longitudinal relaxation time (*T*_1_ value) and normalized magnetization transfer ratio (MTR) were compared between none-to-mild and moderate-to-severe fibrotic bowel walls confirmed by pathological assessments. The results showed that the correlation between the *T*_1_ value and fibrosis (*r* = 0.438, *p* < 0.001) was lower than that between the normalized MTR and fibrosis (*r* = 0.623, *p* < 0.001). Overall, the *T*_1_ values (*t* = −3.066, *p* = 0.004) and normalized MTRs (*z* = 0.081, *p* < 0.001) in none-to-mild fibrotic bowel walls were lower than those in moderate-to-severe fibrotic bowel walls. The area under the curve (AUC) of the *T*_1_ value (AUC = 0.716, *p* = 0.004) was significantly lower than that of the normalized MTR (AUC = 0.881, *p* < 0.001) in differentiating moderate-to-severe fibrosis from none-to-mild fibrosis (*z* = −2.037, *p* = 0.042). Our results support the view that the *T*_1_ value could be a promising imaging biomarker in grading the fibrosis severity of CD. However, the diagnostic performance of native *T*_1_ mapping was not superior to MTI.

## 1. Introduction

Crohn’s disease (CD) is a progressive and destructive chronic inflammatory bowel disease. More than 30% of patients with CD develop fibrotic strictures over time, resulting in distortion of tissue architecture and intestinal dysfunction with further potential intractable complications, such as intestinal obstruction, perforation, and fistulas [1,2]. Recent studies [3,4,5] have demonstrated that early medical intervention of intestinal fibrosis may prevent the exacerbation of fibrotic strictures, thereby altering or delaying the natural progression of CD. However, when fibrosis progresses to a certain degree, drugs cannot reverse it, and endoscopic or surgical interventions are required [6]. Therefore, accurate quantitative detection of intestinal fibrosis is of upmost importance in deciding the individual treatment plan and improving the prognosis. Nevertheless, there is no consistent consensus yet regarding the precise quantitative diagnosis of bowel fibrosis. Methods for diagnosing and quantifying intestinal fibrosis have long been sought, including imaging biomarkers or metrics.

In histopathology, intestinal fibrosis is a consequence of the chronic inflammation that is characterized by excessive extracellular matrix protein deposition. Among regularly used imaging modalities, it is conceivable that quantitative magnetic resonance (MR) imaging, with the MR parameters reflecting the fundamental biologic properties of tissues [7], has great potential for the evaluation of intestinal fibrosis. Magnetization transfer imaging (MTI) is a contrast mechanism that is sensitive to the concentration of macromolecules in an aqueous physiologic environment, such as collagens in the bowel tissue [8,9], and has been reported as an accurate noninvasive method for the quantitative assessment of bowel fibrosis [10,11,12]. The increasing magnetization transfer ratio (MTR) of bowel walls with the severity of fibrosis allows for distinction of different degrees of fibrosis.

Recently, native *T*_1_ mapping has been reported as an emerging noninvasive MR quantitative technique of fibrosis assessment. It quantitatively measures the longitudinal relaxation time (*T*_1_ value) that reflects the inherent characteristics of fibrotic tissue and depends on the mobility of the molecules in the tissue, which can be related to various biologic factors, such as macromolecule concentration, water content, and other micro-environment conditions [13,14]. *T*_1_ mapping can depict even small variations in *T*_1_ values within tissue and has been demonstrated with the highest priority in myocardial fibrosis [15,16]. It has an excellent sensitivity to identify lesions that may be missed by conventional imaging sequences [17,18]. Although application of *T*_1_ mapping in abdominal lesions is challenging due to the consideration of temporal resolution in the past decade, this paradigm may have changed with the rapid development of imaging technology. Now *T*_1_ mapping has also shown promise in liver [19,20] and renal fibrosis [21,22]. Additionally, a prior study has demonstrated that using *T*_1_ mapping in the bowel is feasible, thus permitting objective evaluation of the physiological changes in actively inflamed CD in an area that suffers from motion problem [23].

The purpose of this study was to explore the role of *T*_1_ mapping in the characterization of bowel fibrosis and compare its diagnostic performance with MTI in an experimental rat model using transmural histopathological finding as the reference standard. Given that intestinal fibrosis is histologically characterized by excessive extracellular matrix protein deposition, which may change the mobility of tissue molecules, we hypothesized that the *T*_1_ value may reflect this histopathological changes and aid in the assessment of the severity of bowel fibrosis in CD with non-inferior diagnostic performance compared to that of MTI.

## 2. Materials and Methods

### 2.1. Animal Model

To decrease the influence of confounding factors and obtain different degrees of bowel fibrosis, we performed this prospective study in a rat model of CD fibrosis. This study was approved by the corresponding institutional ethics review board (approval number: [2018]237). All experiments were performed in accordance with the ethics regulations of animal research.

In this study, the 2, 4, 6-trinitrobenzene sulfonic acid (TNBS)-induced (1 M, 293.17 mg/mL, Sigma Aldrich, St. Louis, MO, USA) CD rat model was chosen because it is considered a reproducible animal model that corresponds to CD in humans [24]. Sprague-Dawley rats were administered 150 mg/kg of TNBS once weekly for either 2, 3, or 4 weeks, to induce differing degrees of bowel fibrosis. The rats were fasted for 12 h before initiating the model while allowing water ad libitum. After anesthetizing them, TNBS and 50% ethanol solution (volume ratio, 1:1) was slowly instilled into the colon using a gavage needle fitted into a 1-mL syringe that was introduced such that the tip was approximately 6 cm proximal to the anus. After instillation, the rats were turned upside down for approximately 1 min to prevent the solution from leaking out. A total of 46 CD rats were used for this study: 14 for 2 weeks, 20 for 3 weeks, and 12 for 4 weeks. Meanwhile, 8 normal rats without TNBS treatment were enrolled in the control group.

### 2.2. Image Acquisition

The rats were scheduled for MR examination 6–8 days after the last enema to avoid acute inflammatory reactions. Before imaging, the rats were fasted for 24 h while water was permitted ad libitum and were anesthetized with an intraperitoneal injection of 2% pentobarbital sodium (30 mg/kg) and intramuscular injection of raceanisodamine hydrochloride (0.1 mg) (Minsheng Pharmaceutical Croup Bozhou Medicine Co., Ltd., Hangzhou, China) to minimize intestinal peristalsis. MR examination was performed using a 3.0 T MR scanner (Magneton Verio, Siemens, Munich, Germany) with a 4-channel animal coil (Shanghai Chenguang Medical Technology Co., Ltd., Shanghai, China).

Axial and sagittal T2-weighed imaging and axial T1-weighted imaging were routinely performed. *T*_1_ mapping images were obtained using a dual flip-angle (2° and 12°) three-dimensional (3D) gradient echo sequence. MTI images was acquired using two gradient-echo data sets with and without the application of an off-resonance pulse. All imaging parameters are summarized in Table 1.

### 2.3. Image Analysis

To avoid bias in the measurements, the mid-point of the bowel segment with the most luminal narrowing and/or the most thickening wall on MR images was selected for analysis, using the anus and gross lesions as the positioning landmarks for location-by-location matching between the histopathologic section of the resected bowel and the MR images, by a radiologist (X.L.) with 10 years of experience in gastrointestinal MR imaging. Subsequently, the other radiologist (B.L.) with 6 years of experience in gastrointestinal MR imaging who was blinded to the histopathological information measured the *T*_1_ value and MTR by delineating the region of interest (ROI) on the prelabeled segments on the *T*_1_ maps and MTR maps. The ROIs that cover the layers and entire circumference of the bowel walls in the axial section and inter- and extra-gut components were avoided. In each rat, 1–2 discrete targeted bowel segments (interval >2 cm) were selected according to the extent and severity of the bowel lesion, and each segment measurement was used as an independent data for statistical analysis. MTR of the psoas muscle was also measured on the same section of the MTR map. MTR of the bowel wall was divided by the MTR of skeletal muscle to yield a normalized MTR, which was used for statistical analysis. Three months after the first measurement, the *T*_1_ value and normalized MTR were measured by the same radiologist (B.L.) to evaluate the intra-observer repeatability of the *T*_1_ value and normalized MTR.

*T*_1_ value analysis—quantitative *T*_1_ maps were automatically reconstructed on a voxel-by-voxel basis after data acquisition using the MapIt processing tool (MapIt software, Siemens, Erlangen, Germany). *T*_1_ values were calculated as follows [25]:

(1) $$S=M_{0}\frac{sin\alpha \;\left(1-{℮}^{-TR∕T_{1}}\right)}{1-{℮}^{-TR∕T_{1}}\cos\alpha }$$

The signal intensity (*S*) was determined by the equilibrium magnetization (*M*_0_), longitudinal relaxation (*T*_1_), repetition time (*TR)*, and flip angle (*α*). Therefore, Equation (1) can be reformulated into a linear form as follows:

(2) $$\frac{S}{sin\alpha }={℮}^{-TR∕T_{1}}\frac{S}{tan\alpha }+M_{0\;}\left(1-{℮}^{-TR∕T_{1}}\right)$$

Since TR is constant, different flip angles can establish a series of equations. Considering $\frac{S}{tan\alpha }$ as X, $\frac{S}{sin\alpha }$ as Y, ${℮}^{-TR∕T_{1}}$ as a slope, and $M_{0\;}\left(1-{℮}^{-TR∕T_{1}}\right)$ as an intercept, the equation can be easily solved with a linear least square fit. Therefore, the *T*_1_ value can be calculated by using two or more flip angles. The unit of the *T*_1_ values is millisecond.

MTR analysis—MTR maps were generated by using an in-house MATLAB script (Math Works, Natick, MA, USA). MTR were calculated by using the following equation [10]:

(3) $$\mathrm{MTR}\;=\;\frac{M_{0}-M_{S}}{M_{0}}\;\times \;100\%$$

where *M_s_* and *M*_0_ are the signal intensities acquired with and without the off-resonance pre-pulse saturation, respectively. MTR is dimensionless and is expressed as a percentage.

### 2.4. Histopathologic Analysis

After MR examination, the rats were immediately sacrificed to obtain the bowel tissue sample. The specimens were selected by a radiologist (X.L.) who performed a matched evaluation between the MR images and histologic specimens using the anus and gross lesions as the positioning landmarks. Subsequently, the bowel specimens were fixed with 4% paraformaldehyde, embedded in paraffin, and sectioned with 4-μm-thick slices. Hematoxylin and eosin (HE) and Masson trichrome staining were used for the inflammation score and fibrosis score, respectively. A pathologist (Q.C.), with more than 10 years of experience in gastrointestinal pathology and who was blinded to the MR imaging information, evaluated the degree of fibrosis based on a semi-quantitative scoring system (Table 2) [10].

### 2.5. Statistical Analysis

Statistical analyses were performed using SPSS v20.0 (IBM Inc., Armonk, NY, USA) and MedCalc Statistical Software v15.8 (MedCalc Software bvba, Ostend, Belgium). Continuous variables are expressed as the means ± standard deviation, or the medians (interquartile range) if not normally distributed. Normality of data was evaluated using the Shapiro–Wilk test. All tests were two-sided comparisons and *p* values < 0.05 were considered statistically significant. The intra-class correlation coefficient (ICC) was used to assess the intra-observer repeatability of the *T*_1_ value and normalized MTR. ICC > 0.75, 0.5–0.75, and <0.5 were considered good, moderate and poor agreement, respectively. The correlation between the MR parameter (*T*_1_ value or normalized MTR) and fibrosis score was evaluated by using partial correlation analysis after controlling for the inflammation scores. Additionally, a *t*-test or Mann–Whitney *U* test was performed to compare the differences in *T*_1_ values and normalized MTR values between moderate-to-severe and none-to-mild fibrotic bowel walls. A receiver operating characteristic (ROC) curve analysis was performed, and the area under the curve (AUC) was used to determine the diagnostic accuracy of the *T*_1_ value and normalized MTR for differentiating the different degrees of bowel fibrosis. AUC > 0.85, 0.7–0.85, and <0.7 were considered as high, moderate, and low accuracy, respectively. Fisher’s exact test was performed to verify the association between inflammation and fibrosis, and the odds ratio (OR) was calculated. Logistic regression was performed for establishing a model in grading bowel fibrosis.

## 3. Results

### 3.1. Animal Models Results and Histologic Evaluation

**Figure 1:**
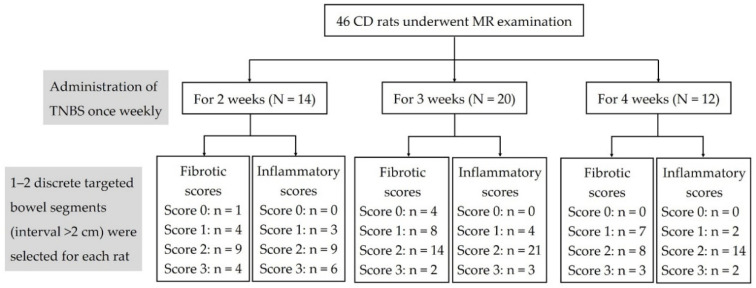
Flowchart of the animal model results and histologic evaluations. CD = Crohn’s disease; MR = magnetic resonance; N = the number of rats; n = the number of bowel specimens; TNBS = 2,4,6-trinitrobenzene sulfonic acid.

In this study, a total of 64 bowel specimens from 46 experimental CD rats were acquired for histopathologic evaluation (Figure 1). Overall, 24 and 40 specimens had none-to-mild and moderate-to-severe fibrosis, respectively, and 9 and 55 specimens had none-to-mild and moderate-to-severe inflammation, respectively. The fibrosis score was associated with the inflammation score (*p* = 0.011, OR = 7.824), thus suggesting one should consider the effects of coexisting inflammation when analyzing the diagnostic accuracy of the *T*_1_ value and normalized MTR in grading bowel fibrosis. In addition, taking the eight normal specimens as the control group, the inflammatory and fibrotic scores were 0.

### 3.2. Diagnostic Efficacy of T_*1*_ Value and Normalized MTR in Characterization of Bowel Fibrosis

**Figure 2:**
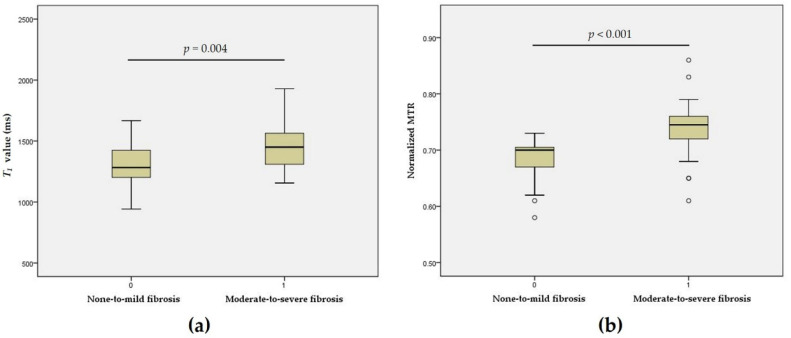
Boxplots showing differences in the *T*_1_ value and normalized MTR between none-to-mild and moderate-to-severe fibrosis. Significant differences in *T*_1_ values (*t* = −3.066, *p* = 0.004) (**a**) and normalized MTR (*z* = 5.081, *p* < 0.001) (**b**) were found between the none-to-mild and moderate-to-severe fibrosis groups. MTR = magnetization transfer ratio; *T*_1_ = longitudinal relaxation time.

In 64 bowel specimens from 46 experimental CD rats, a moderate correlation between the *T*_1_ value and fibrosis score was observed after controlling for the inflammation scores (*r* = 0.438, *p* < 0.001). The *T*_1_ value of none-to-mild fibrosis 1314 ± 180 ms was significantly lower than that of moderate-to-severe fibrosis 1458 ± 183 ms (*t* = −3.066, *p* = 0.004). A good correlation was observed between the normalized MTR and fibrosis score after controlling for the inflammation scores (*r* = 0.623, *p* < 0.001). The normalized MTR of the none-to-mild fibrosis 0.68 (0.040) was significantly lower than that of the moderate-to-severe fibrosis group 0.74 (0.040) (*z* = 5.081, *p* < 0.001) (Figure 2).

The *T*_1_ value and a normalized MTR of the eight specimens from the control group were 1191 ± 204 ms and 0.65 ± 0.06, respectively. Due to the unreliable measurements of the MR parameters on the thin bowel walls of the normal rats, the statistical analysis in this study did not include the data of these eight specimens.

**Figure 3:**
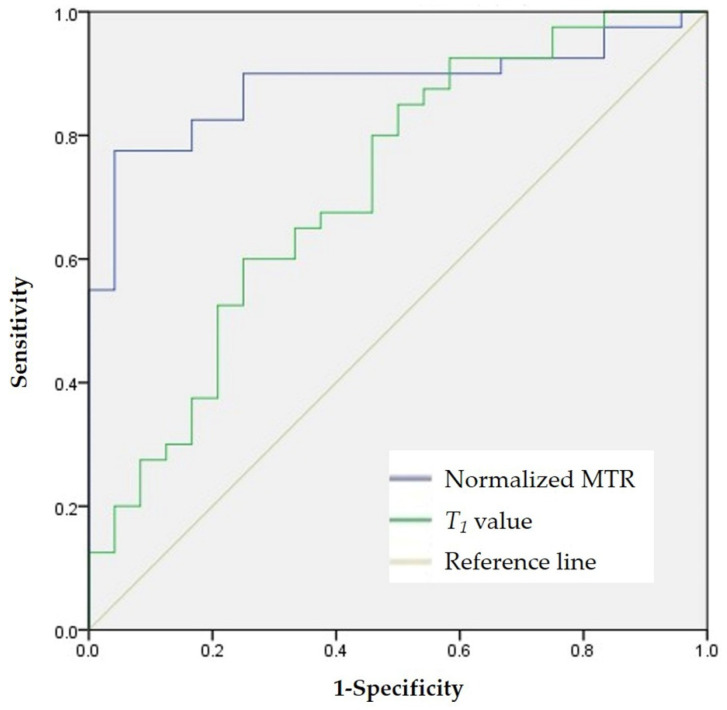
ROC analysis for differentiating none-to-mild fibrosis from moderate-to-severe fibrosis. ROC analysis shows that *T*_1_ value (AUC = 0.716, *p* = 0.004) has moderate accuracy in distinguishing none-to-mild fibrosis from moderate-to-severe fibrosis in all 64 specimens, while normalized MTR (AUC = 0.881, *p* < 0.001) shows good accuracy in differentiating the degree of bowel fibrosis; the difference between the *T*_1_ value and normalized MTR is significant (*z* = −2.037, *p* = 0.042). AUC = area under the curve; MTR = magnetization transfer ratio; ROC = receiver operating characteristic; *T*_1_ = longitudinal relaxation time.

**Figure 4:**
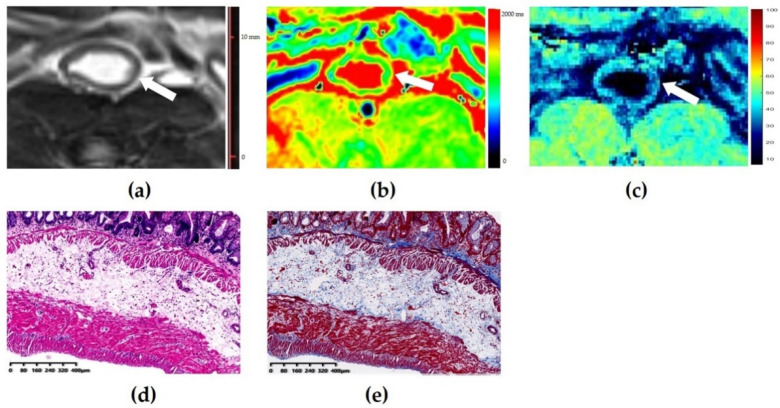
Images of a mildly fibrotic and moderately inflamed bowel wall of a rat. (**a**) Axial T2-weighted imaging reveals a thickened bowel wall. (**b**) *T*_1_ map shows that the *T*_1_ value is 1304 ms (higher than the cutoff value of 1266 ms), thus indicating the presence of moderate-to-severe fibrosis. (**c**) The normalized magnetization transfer ratio (MTR) of the corresponding bowel wall is 0.68 (lower than the cutoff value of 0.72), which suggests the presence of none-to-mild fibrosis. (**d**) Hematoxylin and eosin (magnification = 4.93) and (**e**) Masson’s trichrome staining (magnification = 4.86) depicts moderate inflammation (score = 2) and mild fibrosis (score = 1). In this case, the diagnosis performance of the *T*_1_ value is inferior to that of normalized MTR.

**Figure 5:**
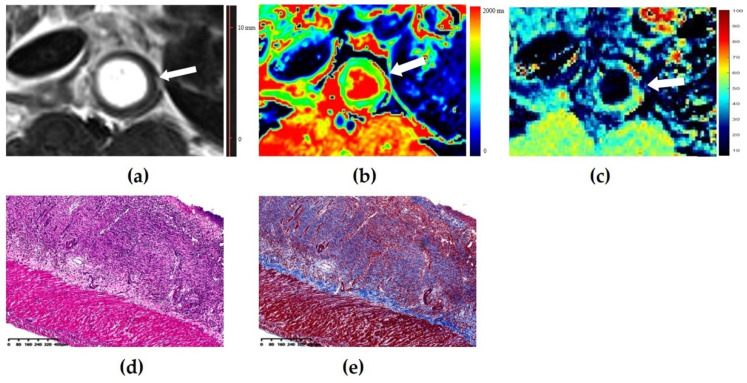
Images of a severely fibrotic and severely inflammatory bowel wall of rat. (**a**) Axial T2-weigheted imaging reveals a markedly thickened bowel wall. (**b**) The *T*_1_ map shows that the *T*_1_ value is 1549 ms (higher than the cutoff value of 1266 ms), thus indicating the presence of moderate-to-severe fibrosis. (**c**) The normalized magnetization transfer ratio (MTR) of the corresponding bowel wall is 0.83 (higher than the cutoff value of 0.72), which suggests the presence of a moderate-to-severe fibrosis. (**d**) Hematoxylin and eosin (magnification = 4.92) and (**e**) Masson’s trichrome staining (magnification = 4.96) depicts severe inflammation (score = 3) and severe fibrosis (score = 3). In this case, the diagnosis performance of the *T*_1_ value is equal to that of normalized MTR.

The *T*_1_ value had moderate accuracy (AUC = 0.716; 95% confidence interval (CI): 0.583–0.848; *p* = 0.004) in distinguishing none-to-mild from moderate-to-severe fibrosis. Using a *T*_1_ value of 1266 ms as the cutoff value, the sensitivity and specificity were 0.850 and 0.500, respectively. High accuracy of the normalized MTR was observed with an AUC of 0.881 (95% CI: 0.795–0.967, *p* < 0.001) in differentiating none-to-mild and moderate-to-severe fibrosis. Using a normalized MTR of 0.72 as the cutoff value, the sensitivity and specificity were 0.775 and 0.958, respectively. There was a significant difference in the AUCs of the *T*_1_ values and normalized MTRs in discriminating varying degrees of bowel fibrosis (*z* = −2.037, *p* = 0.042) (Figure 3). Figure 4 and Figure 5 show images of representative rats with none-to-mild fibrosis and moderate-to-severe fibrosis, respectively.

### 3.3. Effects of Coexisting Bowel Inflammation on the Diagnostic Performance of the T_*1*_ Value and Normalized MTR in Bowel Fibrosis

In bowel segments with moderate-to-severe inflammation (*n* = 55), the AUC of the *T*_1_ value (AUC = 0.684, 95% CI: 0.529–0.840, *p* = 0.030) for differentiating moderate-to-severe fibrosis was significantly lower than that of normalized MTR (AUC = 0.882, 95% CI: 0.792–0.973, *p* < 0.001) (*z* = 2.160, *p* = 0.031). In bowel segments with none-to-mild inflammation (*n* = 9), the AUCs of the *T*_1_ value and normalized MTR in differentiating different grades of fibrosis were 0.714 (95% CI: 0.359–1.000, *p* = 0.380) and 0.643 (95% CI: 0.107–1.000, *p* = 0.558), respectively, which were not significantly different (*z* = 0.218, *p* = 0.827). There were no statistical differences in the AUCs of the *T*_1_ value (*z* = 0.152, *p* = 0.879) and normalized MTRs (*z* = −0.864, *p* = 0.388) in diagnosing bowel fibrosis in the presence of none-to-mild and moderate-to-severe inflammation.

### 3.4. Model of Multivariate Logistic Regression for Grading Bowel Fibrosis

In our study, the *T*_1_ value (*p* = 0.010, OR = 4.130, 95% CI: 1.413–12.074) and normalized MTR (*p* = 0.001, OR = 7.231, 95% CI: 2.312–22.618) from 64 bowel specimens were identified as valuable parameters used to establish a logistic regression model (χ^2^ = 31.026, *p* < 0.001) for grading bowel fibrosis. Its sensitivity, specificity, positive predictive value, negative predictive value, and percentage accuracy in diagnosis of bowel fibrosis were 90.0%, 75.0%, 85.7%, 81.8%, and 84.4%, respectively.

### 3.5. Intra-Observer Agreement

Moderate intra-observer agreement was observed with a *T*_1_ value with an ICC of 0.718 (95% CI: 0.576–0.819, *p* < 0.001). The intra-observer agreement of the normalized MTR was good with an ICC of 0.822 (95% CI: 0.719–0.889, *p* < 0.001).

## 4. Discussion

To the best of our knowledge, our study is the first to date to use native *T*_1_ mapping for assessment of bowel fibrosis of CD and compare its diagnostic efficacy with that of MTI. Our results demonstrated that native *T*_1_ mapping could be a potential noninvasive imaging tool in the characterization of CD bowel fibrosis; however, its diagnostic performance might not be superior to that of MTI.

Native *T*_1_ mapping is a novel MR quantitative technique that provides tissue characterization in vivo and is well known for the detection fibrosis of myocardiopathy [15]. A prolonged native *T*_1_ value has been observed in patients with hypertrophic cardiomyopathy even in the absence of regionally apparent late gadolinium enhancement and hemodynamic obstruction [17]. Nakamori et al. demonstrated that the native *T*_1_ value could predict the histological collagen volume fraction in the myocardium [16]. Recently, the application of *T*_1_ mapping has been prevalent in assessing the fibrosis of some abdominal organs. It was reported that, in staging liver fibrosis, native *T*_1_ mapping yielded a similar high accuracy as acoustic radiation force impulse elastography [19] but had a lower accuracy than MR elastography [26]. In assessing renal fibrosis, the native *T*_1_ value demonstrated a stronger correlation with both alpha-smooth muscle actin expression and Masson’s staining than the apparent diffusion coefficient values [22]. Similar to the results of these studies, our findings demonstrated the feasibility of *T*_1_ mapping in assessing the grade of bowel fibrosis in an animal CD model.

Histopathologically, CD bowel fibrosis is characterized by excessive extracellular matrix protein deposition, which may result in a high *T*_1_ value. In our study, the *T*_1_ value of the fibrotic bowel wall was moderately positively correlated with the fibrosis score. The *T*_1_ value differs significantly with the degree of bowel fibrosis in CD. Furthermore, ROC analysis indicated that using 1266 ms as the cutoff value for moderate-to-severe fibrosis yielded a relative high sensitivity but low specificity. In CD, inflammation and fibrosis always coexist in the bowel wall and both pathological factors could increase the *T*_1_ value. A previous study reported that inflammation interferes with the evaluation of the *T*_1_ value in liver fibrosis and that the *T*_1_ value alone is not accurate in evaluating liver fibrosis [13]. Therefore, it is necessary to evaluate the effectiveness of the *T*_1_ value in assessing bowel fibrosis of varying degrees of inflammation. Our results demonstrated that the AUCs were 0.714 and 0.684 in distinguishing the degree of fibrosis in regions of none-to-mild and moderate-to-severe inflammation, respectively. These findings demonstrate a trend of decrease diagnosis performance, which is expected, although there was no statistically significance between the two AUCs. Considering the sample of none-to-mild inflammation segments was relatively small, further research is needed to derive evidence-based conclusions.

MTI has been proposed as a reliable and accurate imaging technique in distinguishing varying degrees of bowel fibrosis because it is not affected by the severity of inflammation. Image contrast enhancement in MTI is mainly determined by the fraction of macromolecules, such as collagens, in the tissue [8,9]. Due to collagen deposition in the bowel tissue, the more severe the fibrosis, the higher is the normalized MTR. Consistent with the results of our study, a good correlation of normalized MTR with fibrosis scores were observed, thus allowing for differentiation between none-to-mild and moderate-to-severe fibrosis in bowel walls with an AUC of 0.881. Furthermore, we compared the diagnostic performance of *T*_1_ mapping and MTI, and the results revealed that the ability of the *T*_1_ value in differentiating varying severity of bowel fibrosis was not superior to that of normalized MTR. The *T*_1_ value had higher sensitivity but lower specificity in assessing bowel fibrosis when compared with those of normalized MTRs. Both inflammation and fibrosis in the bowel wall could prolong the *T*_1_ value [13], which may partly explain the high sensitivity and low specificity of *T*_1_ mapping. While MTI is a dipolar process that allows chemical exchange between water molecules and macromolecule protons, MTR is mainly determined by the fraction of macromolecule in tissue, such as the deposition of collagen in the case of CD-related fibrosis [8,9,27]. Therefore, MTI is not as sensitive to inflammation, and the image contrast enhancement in MTI may not be observed when the concentration of collagen is low. These reasons might have caused the low sensitivity and high specificity of MTI in evaluating bowel fibrosis.

Therefore, *T*_1_ mapping is more sensitive while MTI is more specific for assessing bowel fibrosis of CD; *T*_1_ mapping may be helpful in early detecting of bowel fibrosis, theoretically. Moreover, our logistic regression model with the *T*_1_ value and normalized MTR had a higher sensitivity than any of the two parameters and an even higher specificity than that of the *T*_1_ value. Hence, a combination of the *T*_1_ value and normalized MTR may have higher efficacy for grading bowel fibrosis in CD.

Repeatability of the measured *T*_1_ values is an important determinant of their clinical utility. Excellent inter-/intra-observer agreement of native *T*_1_ value measurements were observed in the liver, kidneys, and other solid tissues [19,21,22,28]. However, in our study, moderate intra-observer agreement was observed for the a native *T*_1_ value, which was lower than that of the normalized MTR. One possible reason might be that the previous studies performed *T*_1_ mapping using an inversion-recovery-based sequence, which has excellent precision and is highly reproducible [29], while we chose a dual flip-angle 3D gradient-echo sequence, which has a relatively lower spatial resolution despite the faster scanning speed. Additionally, the bowel is likely to be more sensitive, with artifacts based on its relatively limited thickness and motion problems. However, with the development of MR technology, the increasing temporal resolution will work this problem out. Thus, application *T*_1_ mapping to clinical assessment of CD is within our reach.

This study had certain limitations. First, in this preliminary study, we included an animal model rather than patients with CD. Ideally, histologic evaluation of bowel fibrosis should be observed using a full-thickness bowel tissue, which is not available except in surgical cases. However, patients with CD who undergo surgery usually have moderate-to-severe fibrosis, which may result in selection bias. Additionally, the ability of *T*_1_ mapping in characterizing bowel fibrosis can be more accurately assessed in an animal study because the bowel specimen can be obtained immediately after MR imaging, whereas, in patients with CD, the time interval might be several days or weeks. Nevertheless, the effectiveness of *T*_1_ mapping in the diagnosis of intestinal fibrosis needs to be further verified in human studies. Second, due to the unreliable measurements of the MRI parameters on the thin bowel walls of the normal rats, the utility of *T*_1_ mapping in a normal bowel needs to be explored in CD patients in the future. Third, B1 field inhomogeneity may have affected the *T*_1_ value and MTR in our study. Ideally, a single slice with B1 mapping correction is the best method. However, to make the data acquisition as efficient as possible, we ensured the animals had similar weights and sizes and maintained constant environmental conditions, which would decrease the potential B1 effects, as previously reported [30]. Moreover, with the unique advantages of *T*_1_ mapping in the heart and liver, beyond bowel lesions, investigating extra-intestinal complications in such organs of CD using *T*_1_ mapping might be an interesting and promising study in the future. 

## 5. Conclusions

Our results supported that the *T*_1_ value could be a promising imaging biomarker in grading the fibrosis severity of CD. Native *T*_1_ mapping has the potential to assess CD bowel fibrosis but its efficacy in diagnosing the fibrosis severity is not as good as that of MTI, especially in cases of coexisting moderate-to-severe inflammation. A combination of the *T*_1_ value and normalized MTR may have a higher efficacy for grading bowel fibrosis in CD.

## Figures and Tables

**Table 1:** MR imaging sequences and parameters.

Parameters	T_2_WI Sagittal	T_2_WI Axial	T_1_WI Axial	*T*_1_ Mapping	MTI
TR (ms)	4000	3200	700	8.6	538
TE (ms)	99	99	15	3.6	4.4
Matrix	180 × 256	180 × 256	180 × 256	192 × 256	286 × 704
FOV (mm^2^)	70 × 100	49 × 70	49 × 70	52 × 70	73 × 179
Voxel size (mm^3^)	0.4 × 0.4 × 2.0	0.3 × 0.3 × 2.0	0.3 × 0.3 × 2.0	0.3 × 0.3 × 2.0	0.3 × 0.3 × 2.0
Thickness (mm)	2.0	2.0	2.0	2.0	2.0
FA (degree)	120	120	150	2, 12	30
Bandwidth (kHz)	203	203	151	210	254
Acquisition time (s)	186	200	195	144	130

FA = flip angle; FOV = field of view; MTI = magnetization transfer imaging; TE = echo time; TR = repetition time; T_1_WI = T1-weighted imaging; T_2_WI = T2-weighted imaging.

**Table 2:** Histologic scores for inflammatory and fibrotic Crohn’s disease.

Score	Inflammation	Fibrosis
0 (none)	No inflammation or distortion	No fibrosis
1 (mild)	Lamina propria inflammation only	Minimal fibrosis in submucosa or subserosa
2 (moderate)	Submucosal foci of inflammation and/or foci of transmural inflammation	Increased submucosal fibrosis, septa into muscularis propria and/or septa through muscularis propria, increase in subserosal collage
3 (severe)	Significant, dissecting, confluent transmural inflammation	Significant transmural scar, marked subserosal collagen

## Data Availability

The data presented in this study are available on request from the corresponding author. The data are not publicly available due to ethical.
